# Correlation Analysis Between Trace Elements and Colorectal Cancer Metabolism by Integrated Serum Proteome and Metabolome

**DOI:** 10.3389/fimmu.2022.921317

**Published:** 2022-06-02

**Authors:** Zhi Zheng, Qingfeng Wei, Xianghui Wan, Xiaoming Zhong, Lijuan Liu, Jiquan Zeng, Lihua Mao, Xiaojian Han, Fangfang Tou, Jun Rao

**Affiliations:** ^1^ Jiangxi Provincial People’s Hospital, The First Affiliated Hospital of Nanchang Medical College, Nanchang, China; ^2^ Jiangxi Cancer Hospital, The Second Affiliated Hospital of Nanchang Medical College, Jiangxi Clinical Research Center for Cancer, Nanchang, China

**Keywords:** colorectal cancer, Mg, cholesterol metabolism, DIA-MS, UPLC/Q-TOF-MS/MS

## Abstract

Colorectal cancer (CRC) is currently the third most common cancer with a high mortality rate. The underlying molecular mechanism of CRC, especially advanced CRC, remains poorly understood, resulting in few available therapeutic plans. To expand our knowledge of the molecular characteristics of advanced CRC and explore possible new therapeutic strategies, we herein conducted integrated proteomics and metabolomics analyses of 40 serum samples collected from 20 advanced CRC patients before and after treatment. The mass spectrometry-based proteomics analysis was performed under data-independent acquisition (DIA), and the metabolomics analysis was performed by ultra-performance liquid chromatography coupled with time-of-flight tandem mass spectrometry (UPLC-TOF-MS/MS). Trace elements including Mg, Zn, and Fe were measured by inductively coupled plasma spectrometry (ICP-MS) analysis. Four of the 20 patients had progressive disease (PD) after treatment, and clinical test results indicated that they all had impaired liver functions. In the proteomics analysis, 64 proteins were discovered to be significantly altered after treatment. These proteins were enriched in cancer-related pathways and pathways participating immune responses, such as MAPK signaling pathway and complement/coagulation cascades. In the metabolomics analysis, 128 metabolites were found to be significantly changed after treatment, and most of them are enriched in pathways associated with lipid metabolism. The cholesterol metabolism pathway was significantly enriched in both the proteomics and metabolomics pathway enrichment analyses. The concentrations of Mg in the serums of CRC patients were significantly lower than those in healthy individuals, which returned to the normal range after treatment. Correlation analysis linked key lipids, proteins, and Mg as immune modulators in the development of advanced CRC. The results of this study not only extended our knowledge on the molecular basis of advanced CRC but also provided potential novel therapeutic targets for CRC treatment.

## Introduction

Colorectal cancer (CRC) is the third most common malignancy and remains the second most deadly cancer all around the world (1, 2). In 2020, there were estimated 1.93 million CRC cases and 0.93 million CRC deaths globally. It is predicted that the global CRC incidence is to be increased to 3.2 million cases in 2040. Among new CRC diagnoses, 20% developed metastasis already, while another 25% with localized tumor would develop metastasis later. The delayed diagnosis leads to the high mortality rate of CRC patients, posing a growing public health challenge. Although genetic factors including germline MLH1 and APC mutations play etiologic roles in CRC development, over half of all cases and deaths are greatly affected by non-genetic risk factors such as cigarette smoking, low physical activity, low nutrition diet, high alcohol consumption, obesity, inflammatory bowel disease, and dysregulated gut microbiota (3).

Trace elements have been reported to be significantly correlated with the mortalities of various cancers including breast, ovary, bladder, lung, and pancreas cancer (4). For example, Lin et al. reported that mercury exposure was a risk factor for CRC; high levels of Hg and Pb may contribute to the occurrence and development of gastrointestinal cancer (5). The main functions of trace elements in human are as enzyme components or catalysts in biochemical reactions, and imbalanced trace elements may induce cell damages, DNA injuries, and overactivation of certain signaling pathways in tumor growth (6). For instance, zinc (Zn) is an essential nutritional element which functions in antitumor immunity through its involvement in cell immunity of T-lymphocytes (7). Magnesium (Mg) is a necessary element and required as a cofactor in over 600 enzymes, controlling cellular homeostasis and metabolic pathways. Low serum Mg concentrations among women were identified to be associated with higher CRC risk in the Atherosclerosis Risk in Communities (ARIC) Study (8). Iron (Fe) is one of the most important and abundant trace elements. A tight association between excess iron and increased cancer risk has been demonstrated in epidemiological studies (9).

Recent advances in “omics” studies including genomics, proteomics, metabolomics, and proteogenomic have greatly expanded our understanding of cancer biomarkers and drivers of tumorigenesis (10–12). Key genes, proteins, metabolites, and their signaling pathways in cancer cells are usually probed for potential therapeutic targets. Proteomics provides high-throughput and simultaneous determination of thousands of proteins in biological samples, and it has been applied in a lot of large-scale clinical studies on various types of diseases including coronavirus disease (COVID-19), cardiovascular diseases, and cancers (13). Corey et al. recently reported an aptamer-based proteomics study of fibrosis and detected 4,783 proteins, of which 16 proteins differed significantly between the disease and control cohorts (14). Further analysis identified the ADAMTSL2 protein and eight other proteins that are closely related to advanced fibrosis in adults with non-alcoholic fatty liver disease (NAFLD). Apart from proteomics, metabolomics is another powerful large-scale study approach that semiquantitatively measures all small-molecule compounds in biological samples. In cancer studies, metabolomics is often used for assessing disease prognosis, prediction, and responses to specific therapies (15). In 2021, Yu et al. conducted an untargeted UHPLC-Q-TOF/MS metabolomic analysis on 123 human serum samples from patients with chronic gastritis or gastric cancer (16). Three metabolites, linoleamide, N-hydroxy arachidonoyl amine, and hexadecasphinganine, were determined to be potential diagnostic biomarkers, indicating that disturbed lipid metabolism may be connected to the development of chronic gastritis or gastric cancer. When compared to single “-omic” analysis, integrative analyses of proteomics and metabolomics would likely gain deeper understanding of the molecular changes in diseases and discover biomarkers for diagnosis and therapy. We previously conducted a combined DIA-based proteomics and UPLC-TOF-MS/MS metabolomics study of cutaneous squamous cell carcinoma tissue samples. Significant protein and metabolite differences between tumor and non-cancerous tissues were uncovered, which are enriched in pathways related to DNA damage responses, apoptosis, autophagy, platelet activation, and protein digestion and absorption (10). The same integrative proteomics and metabolomics approach was also applied to characterize the serum samples of 20 advanced CRC patients at stage III or IV (11). The results extended our understanding on the physiopathology of CRC and revealed novel potential CRC serum biomarkers.

In the present study, proteomics and proteomics analyses were conducted on 40 serum samples from 20 CRC patients before and after treatment. Trace elements in these serum samples were also determined. Correlation analyses among proteins, metabolites, and trace elements in CRC serum samples before and after treatment were performed in order to identify critical regulators (trace elements, proteins, and metabolites) and related pathways that may function as important pathogenic factors in CRC and may be used as new therapeutic targets.

## Materials and Methods

### Subjects

Twenty CRC patients in Jiangxi Cancer Hospital from January 2019 to December 2019 were included in this retrospective study (Rao et al., 2021). All of these 20 patients (N1–N20) were diagnosed with stage III or IV CRC according to the 7th edition of the American Joint Committee on Cancer (AJCC). Therapeutic plans for the 20 patients included cetuximab plus CAPIRI administration, cetuximab plus FOLFOX6 administration, CAPOX administration, bevacizumab plus CAPOX administration, and CAPIRI administration (**Supplementary Table S1**). Meanwhile, the *Yiqi Sanjie* formula was also administered in the treatment according to the crux of the traditional Chinese Medicine (TCM). About 5 ml of serum from each CRC patient before and after treatment was collected and immediately stored at -80°C until analysis. A group of 20 healthy individuals were also recruited as the controls, whose age and gender were matched to the CRC patient group.

### Proteomic Analysis

The high-throughput DIA-based proteomics analysis was performed as in the previous report (11). Two microliters of each serum sample was firstly diluted with lysis buffer containing 8 M urea (Sigma, MO, USA), 100 mM Tris–HCl (pH 8.5, Sigma, MO, USA), 1 mM PMSF, and 1 mM EDTA, and then centrifuged at 15,000g for 15 min at 4°C. The extracted proteins in the supernatant were quantified using a BCA protein assay kit (Bi Yuntian, Shanghai, China). The extracted proteins were reduced and digested with trypsin using the filter-aided sample preparation (FASP) method (Promega, Madison, WI). A NanoDrop 2000 instrument (Thermo Scientific, Waltham, MA, USA) was used for determination of the concentrations of digested peptides by measuring absorbance at 280 nm. Three micrograms of trypsin-digested peptides containing iRT peptides (Biognosys, Schlieren, Switzerland) was analyzed in the DDA analysis mode on a Fusion Lumos Orbitrap mass spectrometer (Thermo Fisher Scientific) connected to an EASY-nLC 1000 system (Thermo Fisher Scientific). The LC gradient elution program and MS data acquisition settings were reported previously (11). Meanwhile, the DIA analysis was done the same with DDA. A resolution of 60,000 over an m/z range of 350 to 1,500 was set in the full scan, followed by a resolution of 30,000 in DIA scans. Collision energy, AGC target, maximal injection time, and 45 variable DIA windows were all the same as in the previous report (11). The identification and quantification of proteins were conducted using Spectronaut Pulsar X 12.0 (Biognosys). All results were strictly filtered with a Q value cutoff of 0.01, and p values were calculated according to the kernel density estimator.

### Metabolomics Analysis

Metabolites in serum samples were extracted with 120 μl of 50% methanol buffer. Ten microliters of each extraction was mixed to prepare five pooled quality control (QC) samples. All tested samples were injected in a random order. A Triple TOF 5600 Plus mass spectrometer coupled to a UPLC system (Sciex, Cheshire, UK) was used in the metabolomics study. The column for the reversed-phase separation and the gradient elution program were the same as in our previous study (11). The injection volume was 4 µl while the UPLC flow rate was 0.4 ml/min. The Q-TOF mass spectrometer was operated in alternate positive and negative ion modes. The XCMS software was employed for metabolite identification and quantification. Online databases such as Kyoto Encyclopedia of Genes and Genomes (KEGG) and Human Metabolome Database (HMDB) were used for annotation of the identified metabolites. An in-house MS^2^ spectrum library of metabolites was also used in the identification of metabolites (11).

### Trace Element Analysis

Mg, Fe, and Zn were measured using an iCAP Qc ICP-MS system (Thermo Scientific, Bremen, Germany). The serum samples were washed thoroughly, soaked in HNO_3_ (Merck, Darmstadt, Germany), and rinsed several times with 18 Ω deionized water (Millipore, Bedford, MA, USA). Afterward, serum samples were wet digested and analyzed according to the procedures described before (17). Statistical analysis (Student’s t-test) was performed using SPSS 17.0 software, while a *p* value ≤ 0.05 indicated significant difference.

### Data Analysis

The proteomics and metabolomics data normalization and analyses were conducted as in a previous report (11). Briefly, all data were normalized by setting the median of each protein (or metabolite) to 1.00, while the missing values (if any) were filled with the minimum value. Significantly changed proteins were determined by a paired non-parametric Wilcoxon test with p-value ≤ 0.05. OmicsBean (http://www.omicsbean.com), a multi-omics data analysis tool, was employed for Gene Ontology (GO) and KEGG pathway enrichment analyses. Principal component analysis (PCA) and partial least square discriminant analysis (PLS-DA) were performed using the SIMCA-P software (v13.0, Umetrics, Malmö, Sweden). PLS-DA is a suitable approach for compositional data that consist of more metabolites (in hundreds) than biological samples (only tens) and serves as one of the most effective strategies for metabolic data analysis. Besides, independent t-tests were performed for determining significantly changed metabolites. Potential biomarkers from the differentially expressed proteins were evaluated by receiver operating characteristic (ROC) analysis *via* the MetaboAnalyst web server (http://www.metaboanalyst.ca). R statistical software was used for the correlation analysis. The corresponding *p*-values were determined according to the cor.test function, while *p* values were adjusted in order to control FDR (false discovery rate).

## Results

### The Pathologic and Clinical Data

**Figure 1:**
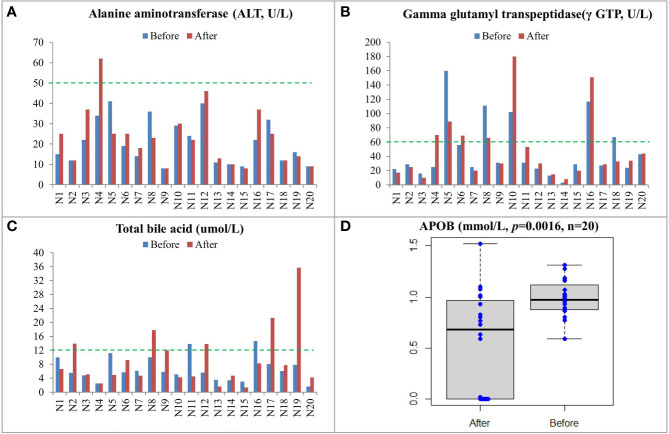
Biochemical examinations and lipid measurements of CRC patients before and after treatment including ALT **(A)**, γ GTP **(B)**, total bile acid **(C)**, and APOB **(D)**. The upper limit of normal level for each item was labeled by a green line.

The clinical information of the 20 recruited patients was listed in **Table 1**. All patients were diagnosed with CRC at stage III or IV. CEA and CA 19-9 levels of the patients were measured. As shown in **Supplementary Table S1**, four patients, namely, N4, N9, N10, and N17, had progressive disease (PD) after treatment, while the rest 16 patients remained with stable disease (SD) after treatment. Biochemical examinations were performed on the 20 patients (**Supplementary Table S2**). Increased levels of gamma glutamyl transpeptidase (γ GTP) and alanine aminotransferase (ALT) in patients N4 and N10 after treatment indicated that they had damaged liver functions (**Figures 1A, B**). The concentration of total bile acid in patient N9 was increased to the critical threshold after treatment, and the concentration of total bile acid in patient N17 was also increased nearly four times after treatment (**Figure 1C**). The APOB levels in all patients were significantly decreased after treatment (**Figure 1D** and **Supplementary Table S3**). The concentrations of high-density lipoprotein cholesterol (HDL), low-density lipoprotein cholesterol (LDL), triglyceride (TG), and total cholesterol (T-CHO) were not changed.

**Table 1:** The details of significant correlations related to proteins or Mg.

Element 1	Element 2	r	*p*
PFN1	KRT81	-0.7125	9.06E-04
SFTPB	TCN1	-0.7067	1.04E-03
IGKV6.21	IGLV2.14	0.7002	1.21E-03
MINPP1	KRT81	0.7026	1.15E-03
GPLD1	MASP1	0.7047	1.09E-03
hCG_2039566	ACTB	0.7068	1.04E-03
IGLC7	ICAM2	0.7158	8.37E-04
GSN	MINPP1	0.7201	7.51E-04
PFN1	KIF21A	0.7214	7.27E-04
FLNA	CDH1	0.7453	3.86E-04
CSF1R	SSC5D	0.7463	3.75E-04
SFTPB	IGHV2.5	0.7704	1.84E-04
PFN1	SFTPB	0.7755	1.56E-04
PFN1	IGHV2.5	0.7794	1.37E-04
IGFBP7	KRT14	0.7822	1.25E-04
TAGLN2	KIF21A	0.8076	5.06E-05
KIF21A	IGHV2.5	0.8398	1.30E-05
PFN1	TAGLN2	0.8515	7.36E-06
SFTPB	TAGLN2	0.8823	1.27E-06
TAGLN2	IGHV2.5	0.9092	1.74E-07
Mg	4DN	0.7760	1.53E-04

### Serum Protein Changes in Patients With Advanced CRC After Treatment

In total, 551 proteins were identified in the proteomics analysis, among which 64 proteins were altered after treatment (*p* value < 0.05) (**Supplementary Table S4**). Of the 64 proteins, the levels of 35 proteins including PFN1, CD99, TAGLN2, and KIF21A were increased, and the levels of the other 29 proteins were decreased (0.23 to 0.96 folds) after treatment.

**Figure 2:**
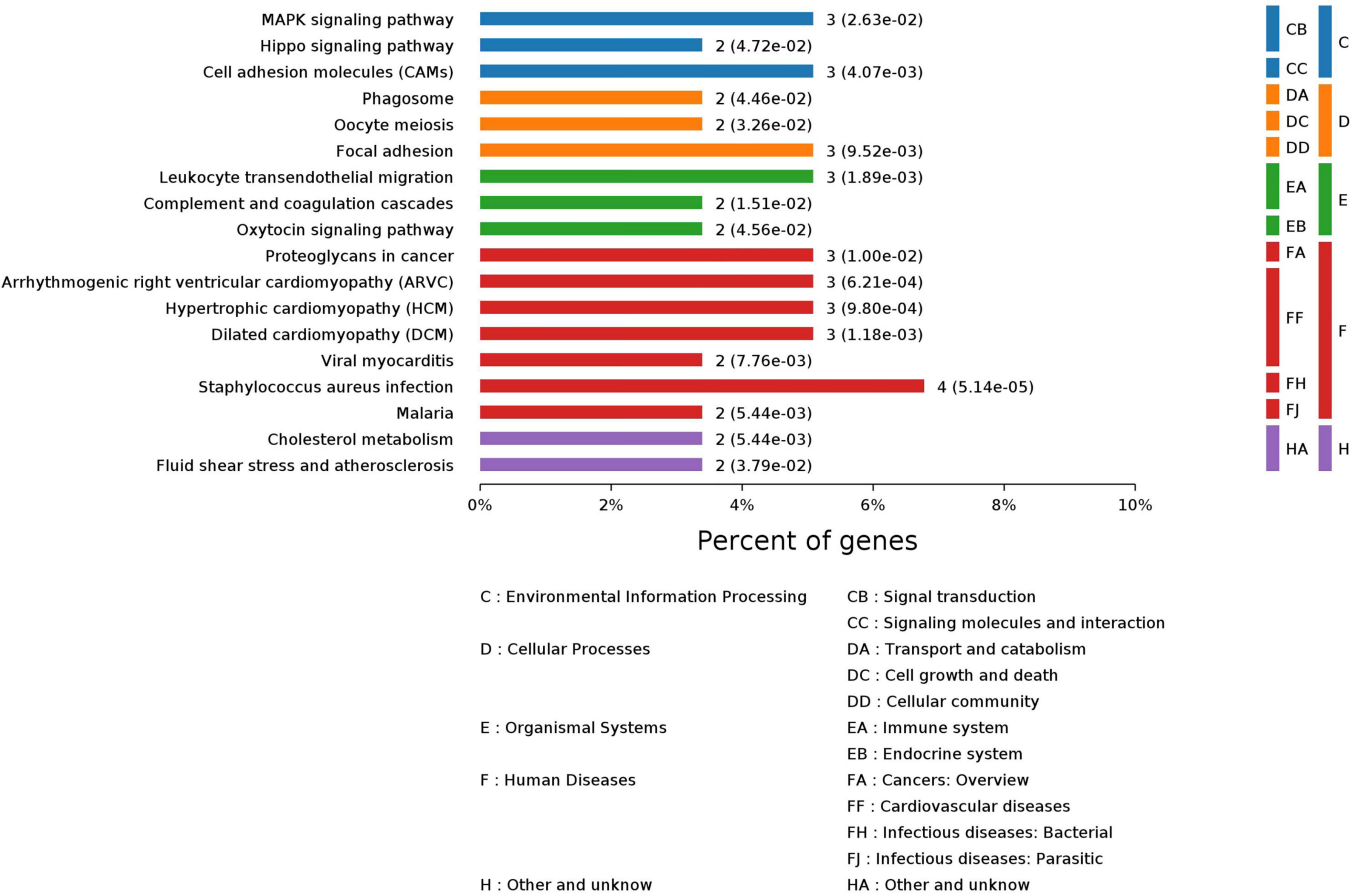
Pathway enrichment analysis of 64 significantly changed proteins in 20 CRC patients after treatment.

GO enrichment analysis of these 64 significantly changed proteins found that 1,133 biological process (BP) terms, 69 molecular function (MF) terms, and 116 cellular component (CP) terms were significantly (*p* values < 0.05) enriched (**Supplementary Tables S5**–**S7**). The top 10 enriched terms for each GO category are shown in **Supplementary Figure S1**. The majorly enriched BP terms were connected to importing into cells, membrane invagination, phagocytosis, engulfment, and adaptive immune response. Immunoglobulin receptor binding, antigen binding, and protein binding were in the top 10 enriched MF terms. The mostly enriched CP terms contained dendritic tree, extracellular space, extracellular region part, extracellular region, and immunoglobulin complex. A pathway enrichment analysis of the significantly changed proteins generated 18 pathways in five functional groups: environmental information processing, organismal systems, cellular processes, human diseases, and other/unknown (**Figure 2**). The largest group, Human Diseases, included *Staphylococcus aureus* infection, arrhythmogenic right ventricular cardiomyopathy (ARVC), malaria, dilated cardiomyopathy (DCM), viral myocarditis, hypertrophic cardiomyopathy (HCM), and proteoglycans in cancer. The “other/unknown” group contained cholesterol metabolism and fluid shear stress and atherosclerosis pathways. The “cellular processes” group had three enriched pathways, namely, focal adhesion, oocyte meiosis, and phagosome. The enriched pathways in the “environmental information processing” group contained pathways related to cancer development such as hippo signaling pathway, cell adhesion molecules (CAMs), and MAPK signaling pathway. The “organismal systems” group contained three pathways in immune or endocrine system: leukocyte transendothelial migration, complement and coagulation cascades, and oxytocin signaling pathway.

### Serum Metabolite Changes in Patients With Advanced CRC After Treatment

**Figure 3:**
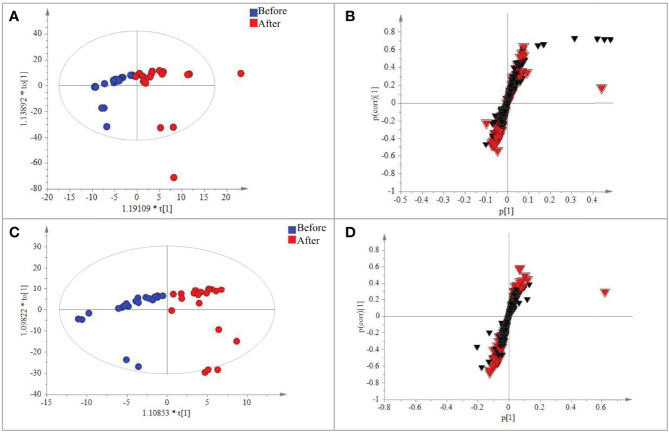
PLS-DA analysis of the metabolic data detected in the positive and negative modes, respectively. **(A)** Plot of the scores from the PLS-DA of metabolic data for CRC patients before and after treatment in the positive negative mode. **(B)** Loading plot from the PLS-DA analysis of metabolic data for CRC patients before and after treatment in the positive–negative mode. **(C)** Plot of the scores from the PLS-DA of metabolic data for CRC patients before and after treatment in the positive–negative mode. **(D)** Loading plot from the PLS-DA analysis of metabolic data for CRC patients before and after treatment in the positive negative mode. The circle dots represent the test serum samples, and metabolites are shown as triangles. Metabolites labeled with the red triangle played important roles for the separation.

**Figure 4:**
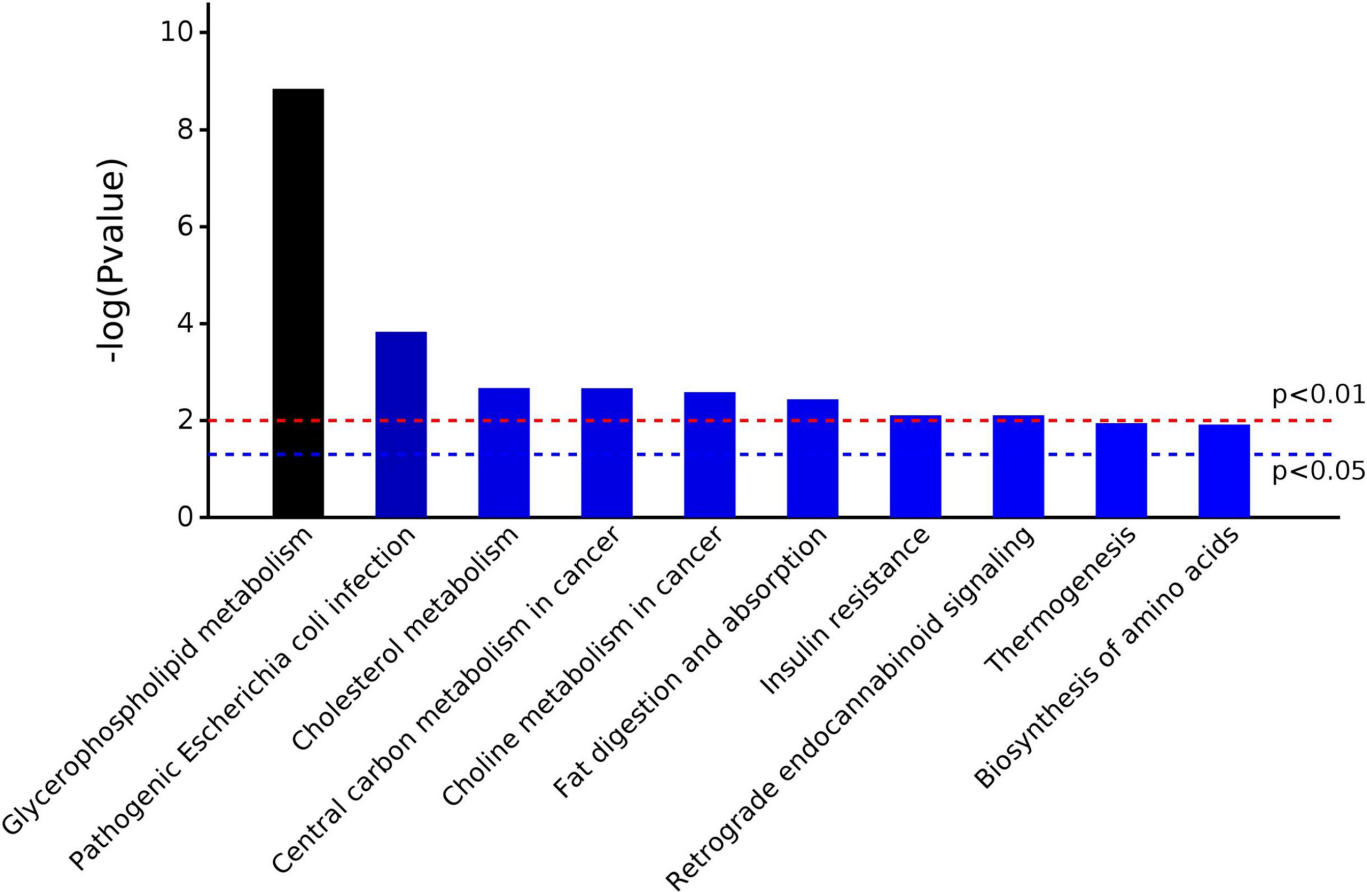
The top 10 significantly enriched pathways in metabolomic analysis.

A total of 567 metabolites were identified in the positive-ion mode, and 431 metabolites were detected in the negative-ion mode. The supervised statistical method PLS-DA was performed on the metabolomics data, and a clear separation between the before and after treatment groups was revealed (**Figure 3**). One hundred twenty-eight metabolites were determined to be significantly changed (0.33- to 2.34-fold) after treatment (**Supplementary Table S8**). Pathway enrichment analysis of these 128 metabolites revealed 20 significantly enriched pathways (*p* < 0.05, **Supplementary Table S9**). Among the top 10 significantly enriched pathways (**Figure 4** **)**, four were related to lipid metabolism such as glycerophospholipid metabolism, cholesterol metabolism, choline metabolism in cancer, and fat digestion and absorption. The cholesterol metabolism pathway was also found to be enriched in the proteomics data analysis, including two proteins (APOB and LRP1) and two metabolites (glycocholate and triacylglycerol). Notably, the change of triacylglycerol determined by metabolomic analysis was constant with that in five items of serum lipid examined by kits, although the latter of which was not significant (*p* > 0.05). The level of APOB protein was identified to be significantly decreased by both proteomics analysis and serum lipid test.

### ICP-MS Trace Element Analysis

**Figure 5:**
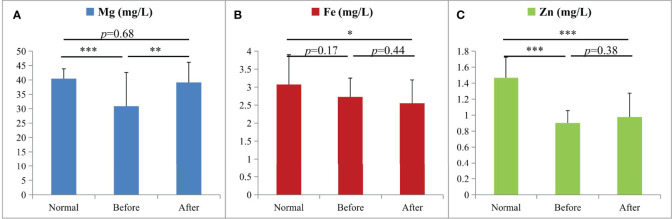
The changes of Mg **(A)**, Fe **(B)**, and Zn **(C)** in ICP-MS analysis among the three groups (*p < 0.05, **p < 0.01, and ***p < 0.001; n = 20 per group): the healthy group (Normal), the group before treatment (Before), and the group after treatment (After).

To investigate the roles of trace elements in CRC, the levels of Mg, Fe, and Zn were determined by ICP-MS in the healthy group (the controls), the group before treatment, and the group after treatment. As shown in **Figure 5**, the concentrations of Mg and Zn in the CRC patients were both significantly lower (*p* < 0.001) than those in the controls. There was no significant change in the Fe concentrations between CRC patients and the controls. After treatment, the levels of Mg and Zn in the CRC patients were increased (*p* < 0.01 and *p* = 0.38, respectively). Especially, the Mg concentrations returned to normal levels (*p* = 0.68) after treatment, while the contents of Zn were still at a significantly lower levels (*p* < 0.001). These results indicated that Mg and Zn may play important roles in the progression and prognosis of CRC.

### Correlation Analysis Among Mg, Proteins, and Metabolites

**Figure 6:**
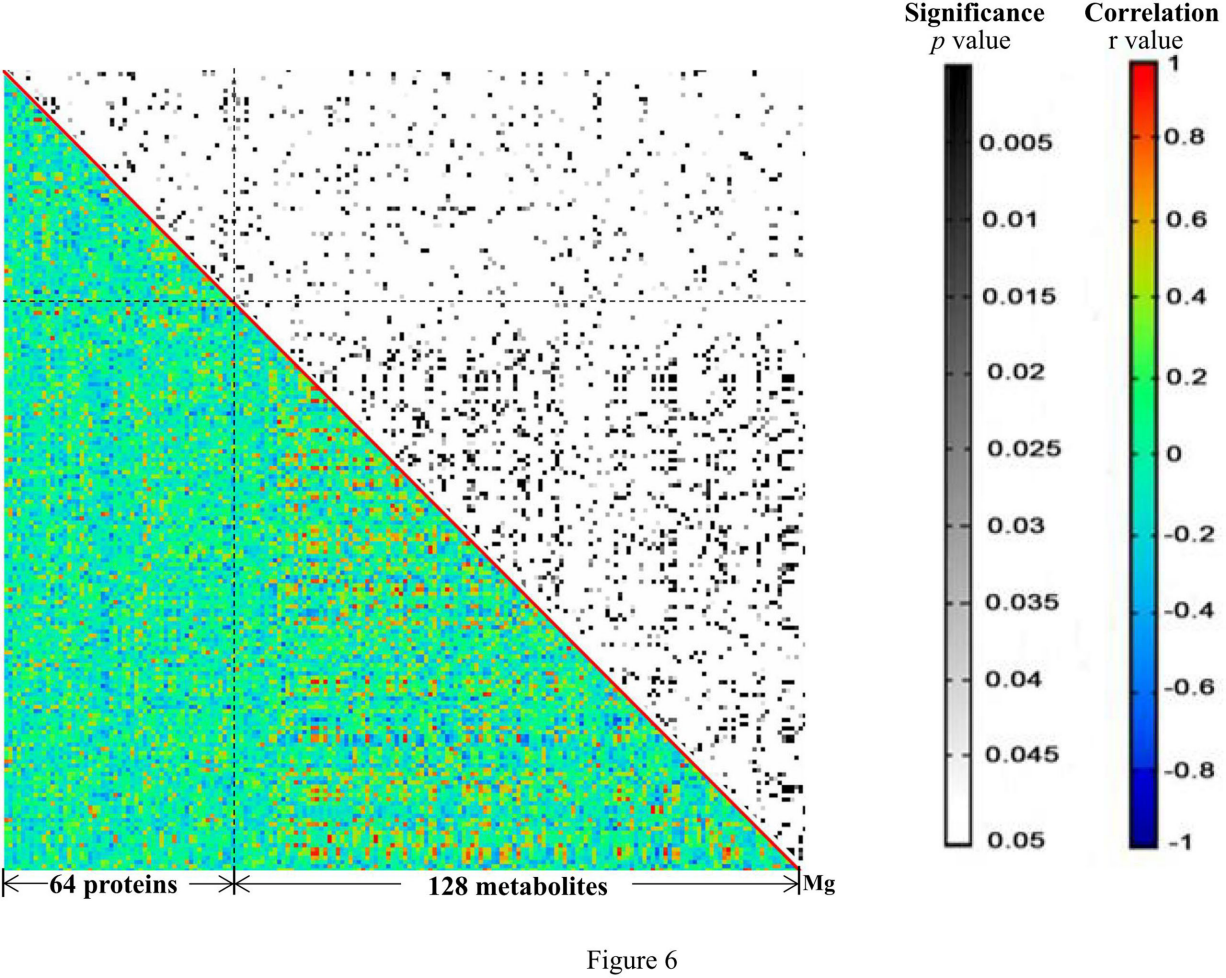
The heat map generated from the results of correlation analysis. The p and r values of the correlations are shown in distinct colors. X- and Y-axes were divided into proteins/metabolites/Mg.

Pearson pairwise correlation analysis was performed among 64 proteins, 128 metabolites, and Mg (**Figure 6** **).** A total of 18,528 correlations were discovered, ranging from -0.7646 for KRT81 and 1-methyladenosine to 0.9896 for (+)-setoclavine and biliverdin. Among the 18,528 correlations, 412 had FDR ≤ 0.05 and r^2^ ≥ 0.49. Only 7 of the 412 correlations were negative correlations, while the rest 405 were positive correlations. Most of the 412 correlations were between metabolites. There were 106 metabolites that were closely associated with each other (359 correlations) or with certain proteins (32 correlations). There were 20 significant correlations (FDR ≤ 0.05 and r^2^ ≥ 0.49) among 24 proteins, and the correlations between TAGLN2 and PFN1, TAGLN2 and SFTPB, and TAGLN2 and IGHV2-5 were all higher than 0.85 (**Table 1**). Mg was found to be only positively correlated with 4′-hydroxy-3′,5,6,7,8-pentamethoxyflavone (r = 0.7760).

## Discussion

The incidence of CRC has dramatically increased in the recent decades, highlighting the significance of understanding its etiology and having effective therapeutic strategies. In the present study, 20 advanced CRC patients at stage III or IV were enrolled to investigate the molecular alterations after treatment. Serum samples were collected and studied by ICP-MS trace element, proteomics, and metabolomics analyses. Most of these advanced CRC patients (80%) had SD after combined traditional Chinese medicine and Western medicine treatment. Hence, the current study aimed to reveal molecular changes of proteins and metabolites after CRC treatment. Serum is one of the most widely used specimens for proteomics and metabolomics investigations, as well as for trace metal research (18). Two high-throughput and non-targeted technologies, DIA-MS and UPLC/Q-TOF-MS/MS, were employed in the proteomics and metabolomics studies. A total of 551 proteins and 719 metabolites were detected, generating large datasets for revealing molecular changes in relation to CRC therapy in a systematic manner.

Increasing evidence has associated impaired liver function to CRC (19). In advanced CRC, liver is the primary metastatic site. Moreover, Mikolasevic et al. (2017) showed a positive correlation between NAFLD and CRC and thus suggested that NAFLD may be a predictor for the development of CRC. In the present study, we found that CRC patients with worsening conditions had impaired liver functions including increased levels of transaminase and total bile acid (20). In the 64 proteins that were significantly changed after treatment, some are associated with liver functions. For example, AOPB and IGF-1 are biomarkers reflecting the grade of liver fibrosis in hepatic patients and their levels were both significantly decreased after treatment. Serum IGF-1 has been reported to be an early indicator of CRC (21, 22). ROC analysis using IGF-1 had a very high AUC value (AUC = 1) to distinguish PD and SD patients, so IGF-1 might be a potential new target in CRC therapy. Moreover, a number of immunity-related proteins such as CR2, CD99, CSF1R, and IGHV2-5 were found to be significantly changed after treatment, and these proteins are involved in adaptive immune response, complement activation, immunoglobulin receptor binding, and antigen binding according to their associated GO terms. In addition, other changed proteins including PFN1 and TAGLN2 were reported to be involved in the development of CRC *via* influencing the immune system (23, 24). TAGLN2 is an oncogenic factor in various types of cancers and may be employed as a potential therapeutic target for CRC. Ding et al. reported that TAGLN2 could regulate the Notch1 signaling pathway by interacting with CD44 and facilitating the proliferation and migration of CRC cells (24). Pathway enrichment analysis of these 64 significantly changed proteins returned several cancer-related pathways and pathways in immune responses.

The UPLC-TOF-MS/MS metabolomics results revealed 128 significantly altered (*p* ≤ 0.05) serum metabolites after CRC treatment. Nearly half of these 128 metabolites are lipids and lipid-like metabolites, among which the level of one liver function-related metabolite, triacylglycerol, was significantly increased after treatment. Pathway enrichment analysis of the significantly changed metabolites returned 20 significantly enriched pathways such as glycerophospholipid metabolism, autophagy, and protein digestion/absorption. Two pathways, insulin resistance and primary bile acid biosynthesis, were re-confirmed to be influenced by treatment. One of the 20 enriched pathways, cholesterol metabolism, was also discovered in the proteomics analysis. This pathway contains APOB, LRP1, glycocholate, and triacylglycerol. It seemed that abnormal cholesterol metabolism may contribute to the progression of CRC. Cholesterol-derived metabolites and proteins which regulate cholesterol metabolism play critical roles in sustaining cancer development and suppressing immune responses (25). Preclinical and clinical studies have already demonstrated that manipulating cholesterol metabolism could inhibit tumor growth, reinvigorate antitumor activity, and thus reshape the immunological landscape. ICP-MS trace element analysis has indicated that Mg and Zn may play key roles in the regulation of CRC development and prognosis (26). Rayssiguier et al. reported that the quantities of Mg and Zn in diet can influence cholesterol metabolism (27). Particularly, Mg plays a significant role in many cellular metabolic reactions, and decreasing of Mg may function as a novel predictive factor of efficacy and outcome of advanced CRC treatment (26). It should be pointed out that some CRC patients in this study were treated with epidermal-growth-factor receptor (EGFR)-targeting antibody Cetuximab, which inhibits cancer proliferation and decreases Mg level as well (28). Lichun et al. reported the positive correlation between the loss of Mg and liver damage and concluded that the serum magnesium concentrations were correlated with certain TCM patterns of symptoms in the process of hepatitis (29). The TCM *Yiqi Sanjie* formula used for CRC treatment in this study probably plays a significant role in affecting magnesium homeostasis also, since some components of the formula such as *Dangshen*, *Astragalus*, and *Lobelia* contain magnesium.

Correlation analysis has been proved to be useful for the discovery of putative key regulatory elements in network regulation (11). There were 412 significant correlations among the significantly changed 64 proteins, 128 metabolites, and Mg. About 98% of the 412 correlations were positive, and most correlations were found between metabolites. Lipids and lipid-like molecules dominated the correlations, indicating their conserved roles in CRC progression and treatment. Meanwhile, several important proteins including KIF21A, TAGLN2, PFN1, and SFTPB were positively correlated with each other (30, 31). For example, the expression level of KIF21A was significantly related to the clinical prognosis outcome of pancreatic ductal adenocarcinoma (PDAC) patients; patients with increased levels of KIF21A had shorter overall survival (OS) time. The SFTPB gene was reported to be a diagnosis marker of mediastinal lung cancer (32). The correlations between every two of KIF21A, TAGLN2, PFN1, and SFTPB were all higher than 0.8, indicating that they play important roles in therapeutic responses of advance CRC. Additionally, there was only one significant correlation involving Mg, which was the positive correlation between Mg and 4′-hydroxy-3′,5,6,7,8-pentamethoxyflavone. Another name for 4′-hydroxy-3′,5,6,7,8-pentamethoxyflavone is 4′-demethylnobiletin (4DN), which is a major metabolite of nobiletin, an immune modulator displaying potential anticancer effects (33). Wu et al. found that 4DN could induce G0/G1 cell-cycle arrest and apoptosis and thus inhibit human colon cancer cell growth (34).

## Conclusions

Altogether, this study investigated the serum molecular changes after treatment of advanced CRC by proteomics, metabolomics, and trace element ICP-MS analysis. The results from the correlation and pathway enrichment analysis revealed important regulatory elements and pathways involved in responses to the treatment of advanced CRC. This work presents new insights into the underlying mechanisms and potential new therapeutic targets for advanced CRC. Nevertheless, these findings need to be further validated due to the limited number of samples used in the current study.

## Data Availability Statement

All of the raw data have been deposited to the ProteomeXchange Consortium *via* the iProX partner repository with the dataset identifier PXD025041.

## Ethics Statement

The studies involving human participants were reviewed and approved by the local ethics committee of Jiangxi Cancer Hospital. The patients/participants provided their written informed consent to participate in this study. Written informed consent was obtained from the individual(s) for the publication of any potentially identifiable images or data included in this article.

## Author Contributions

ZZ, JR, and FT designed the study. ZZ, QW, XW, and XZ carried out the experimental work. LL and JZ prepared the dataset and performed data analyses. LM, XH, and JR wrote the manuscript. All authors contributed to the article and approved the submitted version.

## Funding

This work was supported by grants from the National Natural Science Foundation of China (No. 81960865), Jiangxi Provincial Department of Science (No. 20212BCJL23056 and No. 20212BAG70040), the Excellent Young Scientists Fund of Jiangxi Cancer Hospital (2021EYS01), and National Cancer Center Climbing Fund (No. NCC201814B045). The work was also funded in part by grants from the Science and Technology Plan Project of Jiangxi Provincial Administration of Traditional Chinese Medicine (No. 2019A057).

## Conflict of Interest

The authors declare that the research was conducted in the absence of any commercial or financial relationships that could be construed as a potential conflict of interest.

## Publisher’s Note

All claims expressed in this article are solely those of the authors and do not necessarily represent those of their affiliated organizations, or those of the publisher, the editors and the reviewers. Any product that may be evaluated in this article, or claim that may be made by its manufacturer, is not guaranteed or endorsed by the publisher.
