# Supplementary material for: Correlation Analysis Between Trace Elements and Colorectal Cancer Metabolism by Integrated Serum Proteome and Metabolome
Source: Front Immunol. 2022 Jun 2;13:921317. doi: 10.3389/fimmu.2022.921317 (PMC9201339; doi:10.3389/fimmu.2022.921317)
Supplement: Supplementary file 1 [file DataSheet_1.docx]

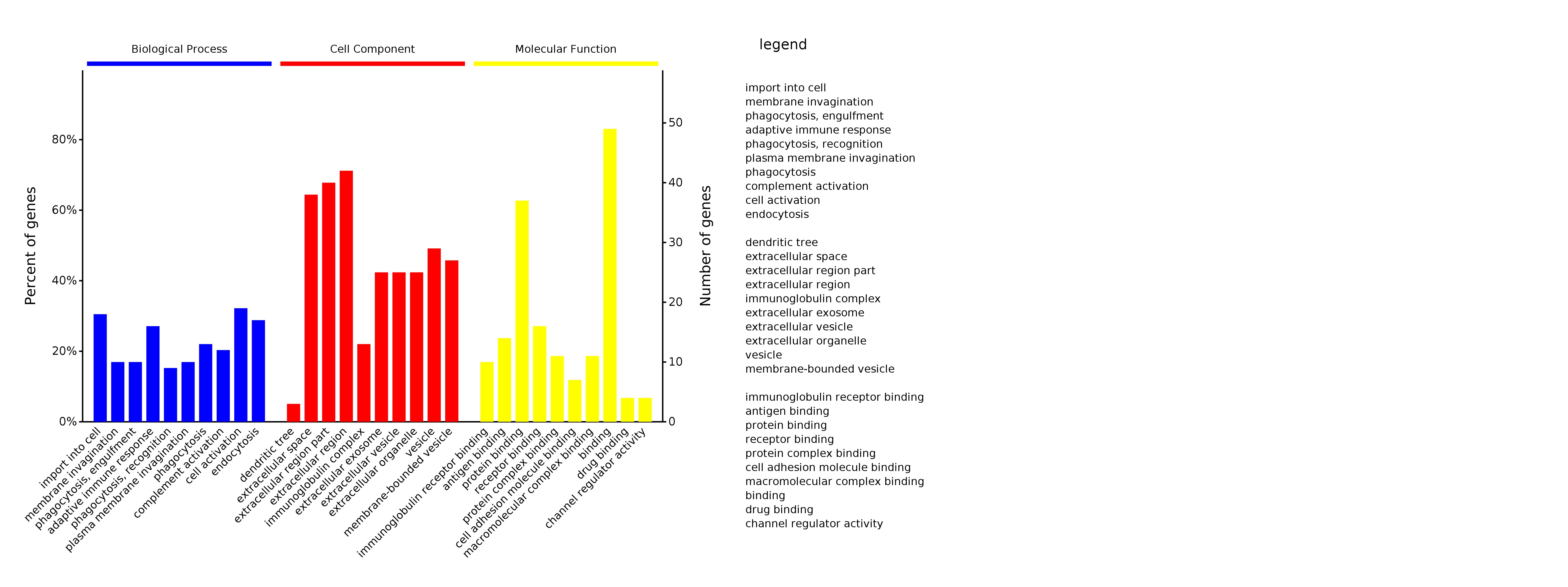


**Figure S1.** The top ten enriched GO terms for each category in proteomic analysis.


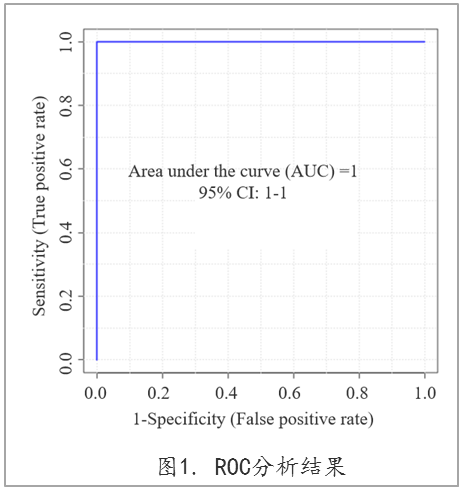


**Figure S2**. ROC curves of IGF1 in 40 tested serum samples from 20 CRC patients before and after treatment.
